# CD73 Expression by CD4 ^+^ T Cells Marks Early Effector Memory T Cells

**DOI:** 10.1111/imm.70011

**Published:** 2025-06-26

**Authors:** Luxia Chen, Sabine Ring, Anna Jurga, Florian C. Kurschus, Alexander Enk, Karsten Mahnke

**Affiliations:** ^1^ Department of Dermatology University Hospital Heidelberg Heidelberg Germany

**Keywords:** 5′ectonucleotidase, CD4^+^ T cells, CD73, memory T cells

## Abstract

CD73 is a membrane bound ectoenzyme, dephosphorylating adenosine mono‐ and di‐phosphate to immunosuppressive adenosine. It is strongly expressed by CD4^+^CD25^+^Foxp3^+^ regulatory T cells, but when analysing conventional CD4^+^ T cells only 50% express CD73. When analysing these two clearly distinct (i.e., CD73^+^ and CD73^−^) populations, we found that the naïve CD73^+^CD4^+^ subset exerted superior proliferation over the CD73^−^CD4^+^ cells, was more resistant to activation induced cells death (AICD) and was prone to develop into a “Th1‐like” cell type, expressing the prototypic cytokines (IFN‐γ, TNF‐α) and specific transcription factors (i.e., *Tbx21*). Upon transfer into lymphopenic hosts, CD73^+^CD4^+^ cells exhibited increased proliferation and survival, and accumulated in inflammatory tissues, developing a CD44^+^CD62L^−^ effector memory phenotype. Therefore, we conclude that CD73 in naïve CD4^+^ T cells functions as a promotor of survival and proliferation of T cells, as well as a marker for their further differentiation into effector T cells.

## Graphical abstract

The role of CD73 in the development of naïve CD4^+^ T cells. Naïve CD4^+^ T cells, which are negative for CD25, CD44, and positive for CD62L, are depicted based on the expression of CD73. CD73^−^ and CD73^+^ subpopulations among naïve CD4^+^ T cells bear distinct characteristics. Upon activation, CD73^+^ T cells secrete more cytokines (IFN‐γ and TNF‐α), and have higher survival and proliferate to a stronger extent. Naive CD73^+^ CD4^+^ T cells preferentially differentiate to Th1 and Th17 cells, whereas more CD73^−^ counterparts polarize to Th2 and iTregs.

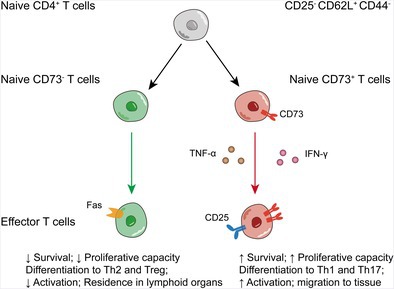

**Abbreviations**
AICD: activation induced cell death
BMDC: bone marrow‐derived dendritic cells
IFN: interferon
IL: interleukin
T_CM_
: central memory T cells
T_EM_
: effector memory T cells
Th: T helper
TNF: tumour necrosis factor
Treg: regulatory T cell

## 1 Introduction

CD73 is a membrane‐bound extracellular 5′ectonucleotidase that converts adenosine di‐ and adenosine monophosphate to adenosine. Due to the immunosuppressive functions of adenosine, functional analysis of CD73 mainly focused on immune regulatory cells. In particular its expression by prototypic CD4^+^CD25^+^ regulatory T cells (Tregs) establishes CD73 as a key marker for Treg subsets, and the production of adenosine was added as a novel immune suppressive mechanism to the pleiotropic suppressive arsenal of Tregs [1]. Recently, this has led to development of anti‐tumour therapies, using anti‐CD73 antibodies to block production of adenosine, establishing CD73 expression as “checkpoint” for tumour treatments [2]. Less data on the function of CD73 beyond its 5′ectonucleotidase activity is available. Initial data by Thompsons group [3, 4] indicate that CD73 may function as T cell co‐stimulatory molecule and others have shown that CD73 acts as adhesion molecule, enabling the adherence and extravasation of T cells to/through the endothelium [5]. However, these data are mostly older than a decade and were not followed up recently.

We initiated this study to investigate the possible functions of CD73 in non‐suppressive cells by analysing its expression in naïve CD4^+^ T cells. These CD4^+^ T cells could be divided into a CD73^+^ and CD73^−^ subset in a 1:1 ratio. When cultivating CD73^+^ and CD73^−^ subpopulations separately, we found that the CD73^+^ subset exerted superior proliferation, was prone to develop into a Th1‐like cell type, had an enhanced tendency to give rise to memory type cells, secreted more proinflammatory cytokines, was more resistant to activation‐induced cell death (AICD) and had a higher survival rate after activation in vitro, as compared to the CD73^−^ subset.

We conclude that CD73 marks an early effector subset within the pool of naïve CD4^+^ T cells. Although the exact mechanisms as to how CD73 brings about the differential functions are not clear, it is of importance as current and future anti‐tumour therapies may use anti‐CD73 antibodies as checkpoint inhibitors. Their possible adverse effects on depleting CD73^+^ effector cells and on the development of immunologic memory have to be carefully examined to establish the beneficial effects of anti‐CD73 antibody treatment regimens in tumour therapies.

## 2 Results

### 2.1 CD73 Expressed by CD4 ^+^Foxp3^−^ Cells Marks a Subpopulation With a Memory Phenotype

**FIGURE 1:**
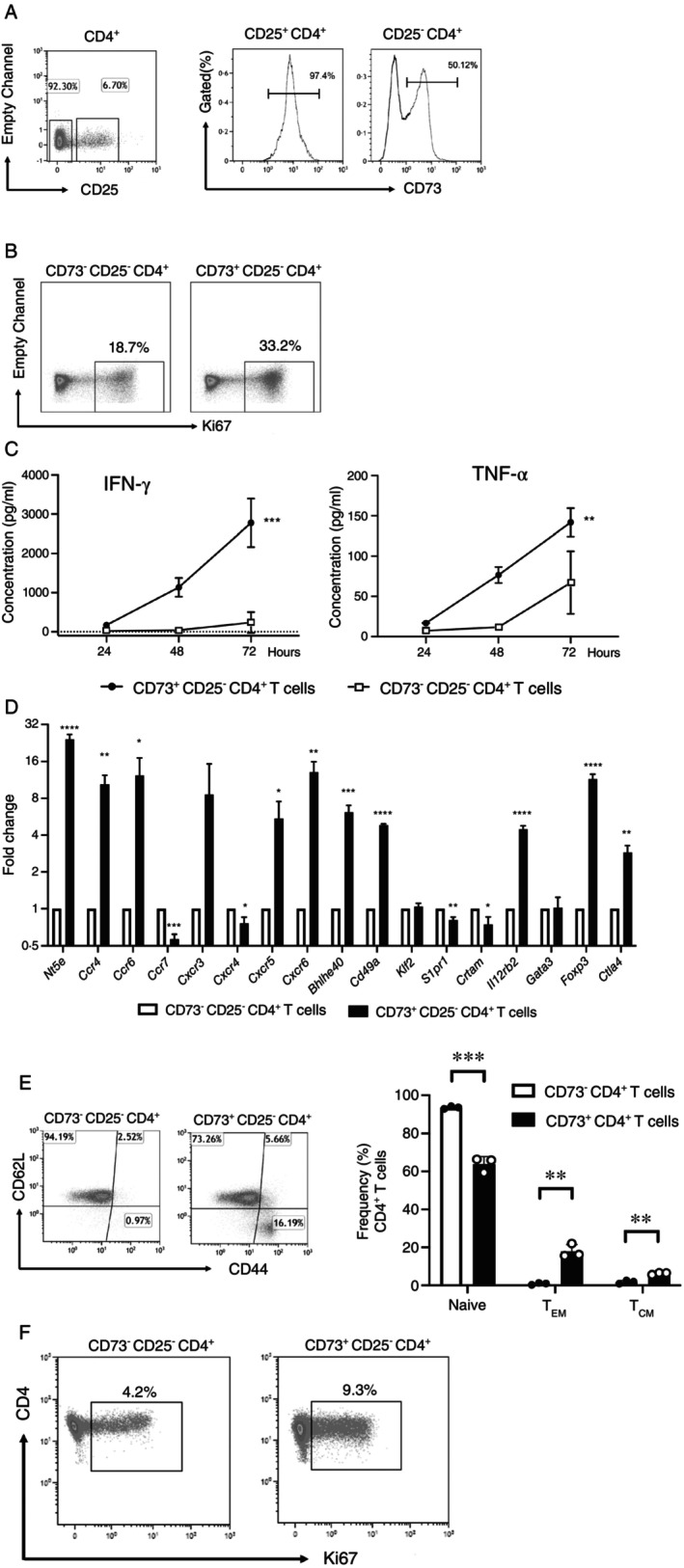
CD73^+^CD25^−^CD4^+^ T cells display superior effector functions over their CD73^−^ counterparts. (A) Expression of CD73 by CD25^+^CD4^+^ T cells and by CD25^−^CD4^+^ T cells in peripheral lymph nodes (pLN) of C57BL/6 mice. (B) Sorted CD73^+^ and CD73^−^CD25^−^CD4^+^ T cells were activated by anti‐CD3/anti‐CD28 mAbs and proliferation was assessed after 3 days by Ki67 staining, shown as a representative dot plot from 3 independent experiments. (C) IFN‐γ and TNF‐α in tissue culture supernatants of cells treated as in (B) were analysed after 24–72 h of culture. Data show mean ± SD (*n* = 3). **p < 0.01 and ***p < 0.001, two‐way ANOVA test. (D) FACS‐sorted cells as in (B) were subjected to qRT‐PCR and gene expression was quantified by using *hprt1* as housekeeping gene. Data are shown as 2^−ΔΔCT^, representing the mean ± SD. *n* = 3, *p < 0.05, **p < 0.01, ***p < 0.001, students' t‐test. (E) Naïve (CD44^−^CD62L^+^), central memory (T_CM_, CD44^+^CD62L^+^) and effector memory (T_EM_, CD44^+^CD62L^−^) cells within the CD73^−^ and CD73^+^ T cells were quantified by FACS. Data show the mean ± SD (*n* = 3). **p < 0.01 and ***p < 0.001, paired t‐test. (F) Sorted CD73^−^ and CD73^+^ T cells were stained intracellularly with Ki67 antibodies. Representative plots of 3 independent experiments depict the expression levels of Ki67.

In mice CD73 is expressed by the majority of Tregs, which are marked by their expression of CD25. Accordingly, in CD4^+^CD25^+^ T cells, 97.4% of the cells express CD73^+^, whereas conventional CD4^+^CD25^−^ T cells divide into CD73^+^ and CD73^−^ cells in a 1:1 ratio (Figure 1A). A division into CD73^+^ and CD73^−^CD4^+^ T cells is already detectable at early levels of T cell development. That is, in the thymus (Figure S1) the expression of CD73 by CD8/CD4 double positive T cell precursors is confined to the TCR^high^ (i.e., mature) subset [6]. Here, 5% of the cells express CD73. In the more mature subset of the single CD4^+^ T cell compartment, which is characterized by being TCRβ^+^CD24^dim^, approx. 18% of the cells express CD73 (Figure S1B), and once T cells have left the thymus, expression of CD73 further increased, as half (56%) of the recent thymic emigrants [7] express CD73 (Figure S1C).

To investigate possible differences in functions of CD73^−^ and CD73^+^ subpopulations in conventional peripheral blood CD4^+^ T cells, FACS‐sorted CD73^+^ and CD73^−^ lineage negative (CD69^−^CD56^−^CD8^−^CD19^−^MHC‐class II^−^CD25^−^) CD4^+^ T cells derived from naïve C57BL/6 mice were activated with anti‐CD3/anti‐CD28 antibodies for 3 days. As indicated by expression of Ki67 (Figure 1B), CD73^+^ T cells proliferated stronger than CD73^−^ T cells. Furthermore, tissue culture supernatants of anti‐CD3/anti‐CD28 antibody‐activated CD73^+^ T cells contained higher concentrations of the pro‐inflammatory cytokines IFN‐γ and TNF‐α, as compared to CD73^−^ cells (Figure 1C). To exclude effects of the anti‐CD73 antibodies used for FACS sorting on T cell proliferation and activation (4), untouched MACS purified T cells were stimulated with PMA/Ionomycin or left alone, in presence or absence of anti‐CD73 antibodies. Here, no differences in proliferation and cytokine production were observed (data not shown).

To determine whether CD73 marks a defined phenotype within conventional CD4^+^ T cells, qRT‐PCR was performed with sorted CD73^−^ and CD73^+^ subpopulations (Figure 1D). Higher expression levels of *Nt5e*, the gene encoding CD73 in T cells, were observed in CD73^+^ cells, whereas the signal was nearly absent in CD73^−^ cells, indicating that FACS sorting resulted in clearly distinct CD73^+^ and CD73^−^ subpopulations. Analysis of the expression of chemokine receptors revealed that CD73^+^ cells show elevated expression of *Ccr4*, *Ccr6*, *Cxcr5*, and *Cxcr6*, as well as a reduced expression of *Ccr7* and *Cxcr4*, which may indicate an increased capacity to migrate to peripheral tissues [8].

CD73^+^ T cells also exhibited a Th1‐like gene patterns, with increased expression of *Il12rb2*, whereas no preferential expression of *Gata3*, a transcription factor associated with Th2 cells, was observed in CD73^+^ T cells. Despite the fact that Tregs are already removed by sorting, higher expression of genes associated with Tregs, such as *Foxp3* and *Ctla4* was recorded in CD73^+^ cells. This may indicate an early precursor‐state of Tregs, given that Tregs have been shown to maintain CD73 expression unequivocally, which is essential for their regulatory function [9].

Moreover, CD73^+^ T cells exhibited high expression levels of *Bhlhe40*, a transcription factor associated with memory T cell differentiation, and displayed substantial expression of the tissue homing molecule *Cd49a*. In line with this, a reduction in *S1pr1* expression, a receptor crucial for T cell activation and tissue retention [10], was observed in CD73^+^ cells. Thus, these data support a receptor expression profile specific for peripheral tissue homing.

The divergent gene expression patterns of CD73^−^ and CD73^+^ cells led us to inquire whether this distinction is already inherent in naïve T cells, therefore, the initial composition of effector memory (T_EM_) and central memory (T_CM_) T cell subtypes within these two populations was examined (Figure 1E). CD73^+^ cells consisted of fewer naïve (CD44^−^CD62L^+^) cells (94.19% vs. 73.26%), more T_EM_ (16.19% vs. 0.97%) and more T_CM_ (5.66% vs. 2.52%) than CD73^−^ cells. This supports our notion that the superior effector functions of CD73^+^ T cells is possibly due to a higher frequency of effector and central memory cells in the CD73^+^ subset. In addition, freshly FACS‐sorted CD73^+^ cells displayed a twofold expression of Ki67 (9.25%) over their CD25^−^CD73^−^ (4.14%) counterparts (Figure 1F), indicating a spontaneous advantage in proliferation of CD73^+^ cells over CD73^−^ cells.

To test inasmuch as our findings in mice also relate to humans, we analysed PBMCs from three different healthy donors for CD73 expression of their different T cell populations. We distinguished naïve, effector memory (T_EM_), central memory T cells (T_CM_) and effector memory T cells re‐expressing CD45RA (T_EMRA_) using CD45RA, CD45RO, CCR7, CD27, CD25 and FoxP3 markers as shown in Figure S2. Similar to previously published data (6), we found that in the CD4^+^ T cell compartment T_EM_ cells contained the highest fraction with 23% CD73^+^ T cells, whereas naïve T cells contained around 17% of CD73^+^ T cells. T_CM_ cells contained the lowest amount of CD73^+^ cells and CD25^+^FoxP3^+^ T cells were on average 16% CD73 positive with a high variation (Figure S2A,C). Of note, in the CD8^+^ T cell compartment, naïve T cells were nearly homogeneously positive for CD73, whereas T_EMRA_ cells showed very little T cells expressing CD73. In T_CM_ and T_EM_ cell populations around 42% and 29% cells expressed CD73, respectively (Figure S2B,C). We also tested for a correlation of CD73 expression with IFN‐γ production upon stimulation with PMA/Ionomycin in the presence of Brefeldin A. First, we found IFN‐γ expression in both CD73‐positive and CD73‐negative T cell populations (Figure S2D). But when we analysed CD73‐positive and CD73‐negative T cell populations separately, we found that CD4^+^ and CD8^+^ T_CM_ and T_EM_ T cell populations which were positive for CD73, indeed showed a higher percentage of IFN‐γ positivity compared to those negative for CD73 (Figure S2E). These IFN‐γ positive T cell populations, as well as CD73^+^CD8^+^ T_EMRA_ cells, were also higher in their level of IFN‐γ expression, shown by the geometric mean fluorescence intensity (Geo MFI) (Figure S2F). Only a tiny fraction of naïve CD4^+^T cells expressed IFN‐γ upon activation. Interestingly, also here the CD73 positive population expressed distinctly higher levels of this Th1 cytokine (Figure S2G). Thus, in mice as well as in humans CD73 may mark a subset of T cells prone to act as effectors.

### 2.2 CD73 ^+^ CD44 ^−^ CD62L ^+^ CD25 ^−^ CD4 ^+^ Cells Highly Express Activated T Cell‐Associated Transcripts and Display Superior Proliferation Advantage

**FIGURE 2:**
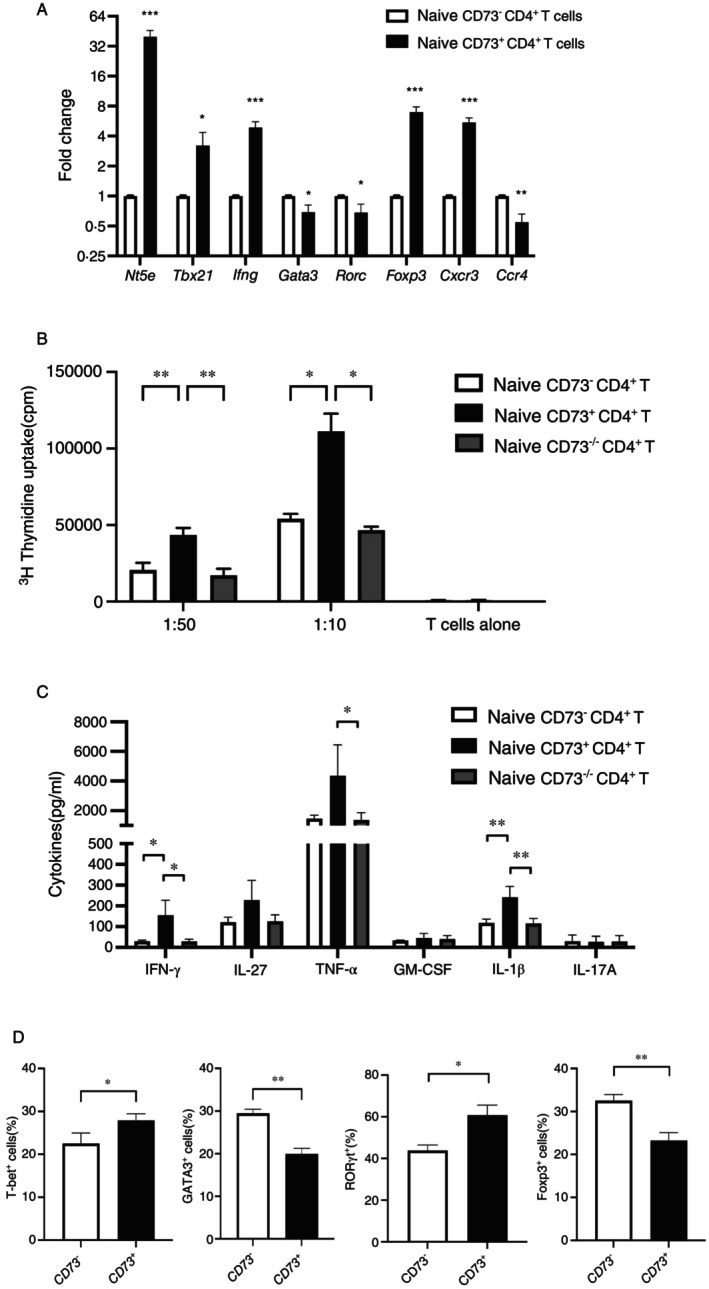
Naive CD73^+^CD4^+^ T cells display Th1 cell type‐like features. (A) Naïve (CD25^−^CD44^−^CD62L^+^) CD4^+^ T cells were FACS sorted according to their expression of CD73 and gene expression was quantified by qRT‐PCR, with *hprt1* as housekeeping gene for normalization. Data are shown as 2^−ΔΔCT^, representing the mean ± SD. *n* = 3, *p < 0.05, **p < 0.01, ***p < 0.001, students' t‐test. (B) FACS sorted naïve CD73^−^ and CD73^+^ CD4^+^ T cells from C57BL/6 mice, as well as naïve CD73^−/−^CD4^+^ T cells from CD73^−/−^ mice, respectively, were cultivated with graded doses of bone marrow derived dendritic cells in presence of anti‐CD3 antibodies. Proliferation was measured after 48 h by ^3^H‐thymidine uptake. Data represents the mean ± SD (*n* = 3). *p < 0.05 and **p < 0.01, two‐way ANOVA test. (C) Secretion of cytokines (as indicated) from BMDC—T cell co‐cultures were analysed after 72 h. Data represents the mean ± SD (*n* = 3). *p < 0.05, **p < 0.01 by one‐way ANOVA with Dunnett's posthoc test. (D) Cells as in (B) were polarized according to standard protocols. After 96 h, cells were harvested and analysed by flow cytometry for marker expression as indicated. Data are shown as the mean ± SD (*n* = 3). *p < 0.05, **p < 0.01, students' t‐test.

To exclude the impact of in vivo differentiation, the function of CD73 in naïve murine CD4^+^ T cells was investigated next. FACS‐sorted, lineage^−^ naïve (CD44^−^CD62L^+^CD25^−^CD4^+^) CD73^−^ and CD73^+^ T cells, were subjected to qRT‐PCR and differential expression of relevant genes was assessed (Figure 2A). The strong expression of the gene for CD73 (*Nt5e*) in CD73^+^ T cells, as opposed to the CD73^−^ subset, confirmed the purity of the sorted populations. Naïve CD73^+^ cells were enriched with T‐helper 1 (Th1) lineage‐specific transcripts, that is, *Tbx21* and *Ifng*, and expressed reduced levels of *Gata3* and *Rorc*. However, expression of *Foxp3*, the transcription factor for Tregs, was found to be higher in naïve CD73^+^ T cells, which is reminiscent of the fact that all fully differentiation Tregs express CD73. As for the expression of chemokine receptors, naïve CD73^+^ cells displayed increased expression of *Cxcr3*, which acts as receptor for the IFN‐γ induced chemokines CXCL9‐11 and is expressed preferentially on Th1 lymphocytes [11] as well.

For functional analysis, FACS‐sorted naïve cell subtypes were stimulated in the presence of bone marrow‐derived dendritic cells (BMDCs) at various ratios for 48 h. Here, and in the following, T cells from CD73 deficient (CD73^−/−^) mice were also used. As shown in Figure 2B, naïve CD73^+^ T cells exhibited a more robust proliferation than naïve CD73^−^ and CD73^−/−^ CD4^+^ T cells at all ratios with BMDCs. Analysis of the cytokines in the supernatants of naïve CD4^+^ T cells after coculture with BMDCs revealed that naïve CD73^+^CD4^+^ T cells produced increased levels of IFN‐γ, TNF‐α, and IL‐1β upon stimulation (Figure 2C). In contrast, the levels of cytokines secreted in the respective cocultures of respective CD73^−^ or CD73^−/−^ CD4^+^ T cells with BMDCs, were comparably lower than those observed in CD73^+^:BMDC cocultures.

CD73^−^ and CD73^+^ cells were further tested in established in vitro polarization regimens to induce Th1, Th2, Th17 and Treg subsets, [12] and the intracellular expression levels of transcription factors such as T‐bet, GATA3, RORγt, and Foxp3, which serve as indicators for Th1, Th2, Th17 and Treg cells, respectively, were assessed (Figure 2D). The data demonstrated increased differentiation of naive CD73^+^ cells into Th1 cells, as indicated by the increased percentage of T‐bet^+^ cells. Conversely, naive CD73^−^ T cells were more likely to polarize towards Th2 cells, which was apparent by higher frequencies of GATA3^+^ cells. Despite harbouring substantial levels of Foxp3 mRNA, naïve CD73^+^ cells generated fewer Foxp3^+^ cells upon polarization towards Treg cells, whereas more RORγt^+^ cells were generated from naive CD73^+^ populations as compared to naïve CD73^−^CD4^+^ T cells under Th17 polarizing conditions. Together with increased production of IFN‐γ, the augmented expression of genes relevant for T cell lineage‐commitment, that is, *Ifng* and *Tbx21* and *Cxcr3*, indicate a preference of CD73^+^ T cells for development into Th1‐like lymphocytes [11], which are prone to give rise to memory T cells [13].

### 2.3 Naïve CD73 ^+^ CD4 ^+^ T Cells Are More Resistant to Cell Death

**FIGURE 3:**
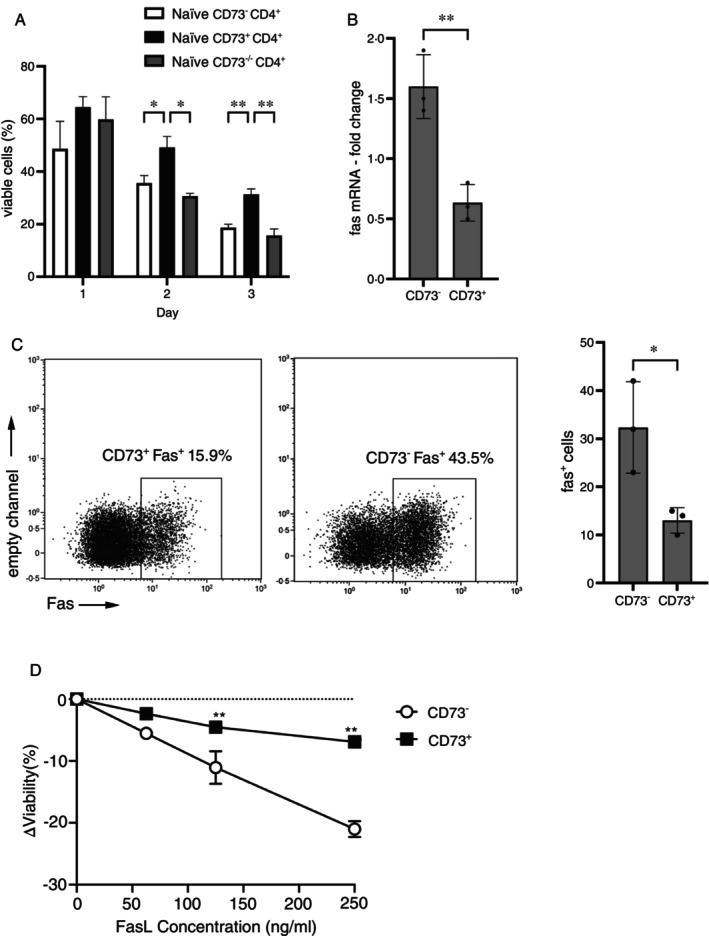
CD73^+^CD4^+^ T cells are more resistant to cell death than CD73^−^ cells. (A) Sorted cells as indicated were stained with Annexin V and zombie aqua and analysed by flow cytometry. The percentages (mean ± SD) (*n* = 3). *p < 0.05, **p < 0.01, two‐way ANOVA of viable cells (negative for Annexin V and zombie aqua) after 24, 48 and 72 h are shown. (B) CD95 mRNA expression was quantified in 24 h‐activated cells by qRT‐PCR. mRNA levels of respective untreated cells were set to 1. Shown are means ± SD (*n* = 3), **p < 0.01, students' t‐test. (C) Cells as in (B) were evaluated for expression of CD95 by flow cytometry. Representative dotplots and means ± SD (*n* = 3), *p < 0.05, students' t‐test, are shown. (D) Sorted CD73^−^ and CD73^+^ cells were cultivated with graded doses of CD95L. After 4 h, viability of cells was measured by flow cytometry (right). Shown are means ± SD (*n* = 3), **p < 0.01, students' t‐test.

Naïve CD73^+^ T cells exhibit enhanced effector functions, which may be directly related to their ability to survive. To address this question, naive CD73^−^, CD73^+^ as well as naïve CD73^−/−^CD4^+^ T cells were activated and their viability was determined by excluding Zombie Aqua positive (dead) and/or Annexin V positive (apoptotic) cells by facs. CD73^+^ T cells survived at higher rates than CD73^−^ and CD73^−/−^ cells (Figure 3A), indicating that expression of CD73 offers an advantage in survival of naïve T cells after activation. As cells may undergo activation‐induced cell death (AICD) induced by stimulation of CD95 (Fas) or TRAILR [14], we next assessed the expression of CD95 by RT‐PCR and flow cytometry in unstimulated and TCR‐stimulated CD4^+^ T cell subsets as indicated (Figure 3B). Upon activation, CD73^−^ cells upregulated mRNA coding for CD95, in contrast, CD73^+^ cells downregulated the respective mRNA. This regulation of CD95 transcription was also reflected by differential expression of CD95 proteins (Figure 3C) by CD73^−^ and CD73^+^ cells, respectively. That is, CD73^+^ cells expressed less CD95 upon activation as their CD73^−^ counterparts. Consequently, CD73^+^ cells were less susceptible to CD95L‐induced cell death in vitro as opposed to CD73^−^ T cells (Figure 3D). Therefore, CD73 may protect CD4^+^ cells from AICD by downregulating CD95.

### 2.4 CD73 Facilitates Spontaneous Proliferation and Activation of naïve CD4 ^+^ T Cells In Vivo

**FIGURE 4:**
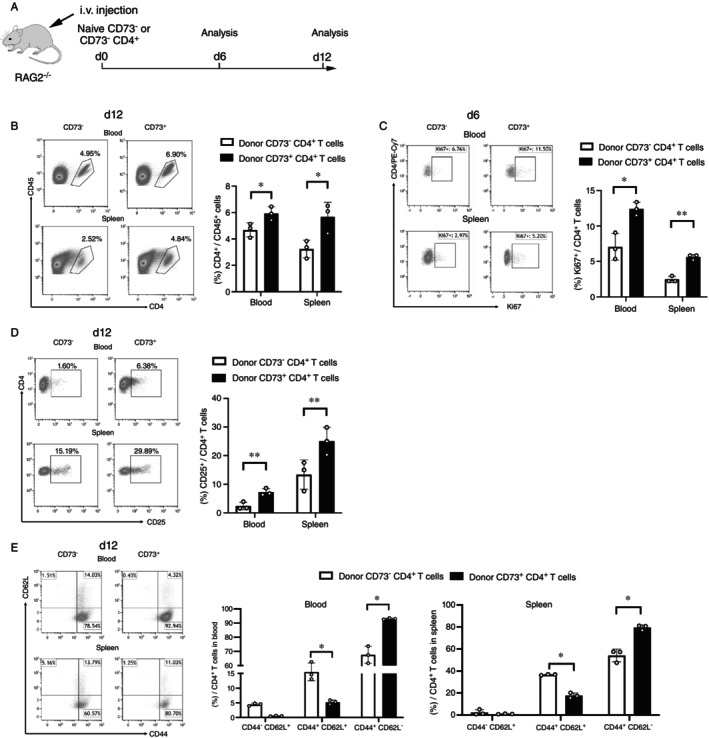
CD73 facilitates proliferation and activation of naïve CD4^+^ T cells in vivo. (A) Schematic of the experimental regimen. (B) The proportion of transferred CD4^+^ cells within the viable CD45^+^ cell population in blood and spleen was assessed on day 12. (C) depicts the frequency of Ki67^+^ cells among the donor cells in blood and spleen on day 6. (D) The prevalence of activated (CD25^+^) T cells within the donor cells in blood and spleen was quantified on day 12. (E) The phenotypic development of transferred cells categorized into naïve (CD44^−^CD62L^+^), T_EM_ (CD44^+^CD62L^−^), and T_CM_ (CD44^+^CD62L^+^) cells was analysed in blood and spleen on day 12. All results are shown as representative dot plots and bar graphs that display means ± SD (*n* = 3). *p < 0.05; **p < 0.01; ***p < 0.005, students' t‐test.

To analyse the role of CD73 for the survival and proliferation of naïve CD4^+^ T cells in vivo, sorted naïve CD73^+^ and CD73^−^ cells were transferred into Rag2^−/−^ recipient mice, and the frequencies of the transferred cells in spleens and blood were analysed by flow cytometry (Figure 4A). As shown in Figure 4B, the frequency of transferred naïve CD73^−^ cells on day 12 in the blood of Rag2^−/−^ mice was lower than that in mice receiving naïve CD73 +^+^ cells, and this difference was also apparent in the spleens. Furthermore, the expression of Ki67 by transferred CD4^+^ T cells on day 6 in the blood and spleen (Figure 4C) was measured to determine their proliferation. In agreement with previous in vitro results, a substantial increase in Ki67 expression in transferred CD73^+^ cells was observed. Next, we analysed whether the increased frequencies of CD73^+^ cells were accompanied by an increased activation by quantifying the expression of CD25 by the donor CD4^+^ T cells following adoptive transfer into Rag2^−/−^ recipient mice. Figure 4D displays an increase in CD25 expression in donor CD73^+^ cells in blood and spleens.

Naïve CD4^+^ T cells proliferate and differentiate directly into memory T cells upon transfer into lymphopenic hosts [14], accordingly, we analysed whether memory T cell differentiation in vivo in Rag2^−/−^ mice is guided by CD73 expression (Figure 4E). 12 days after transferring naïve CD4^+^ T cells to Rag2^−/−^ mice, the donor CD4^+^ T cells, possessing a naïve (CD44^−^CD62L^+^) phenotype before transfer, acquired central memory (CD44^+^CD62L^+^) and effector memory (CD44^+^CD62L^−^) phenotypes. In blood and spleens, the frequency of cells expressing an effector memory phenotype was increased in transferred CD73^+^ cells as compared to CD73^−^CD4^+^ cells. Vice versa, more central memory cells developed from the CD73^−^ T cell subset.

The overall increased survival and higher expression of Ki67 in vivo indicate that CD73^+^ T cells are long‐lived under homeostatic conditions. Additionally, CD73 enhances the differentiation of naïve CD4^+^ T cells into effector memory cells, which is consistent with in vitro RNA expression data. These results are further supported by findings [15], demonstrating that CD73 also favours the homeostatic proliferation of CD8^+^ T cells. Collectively, these data indicate that CD73 supports homeostatic proliferation of naïve CD4^+^ and CD8^+^ T cells in vivo.

### 2.5 CD73 Enhances the Migration of CD4 ^+^ T Cells to Inflamed Skin

**FIGURE 5:**
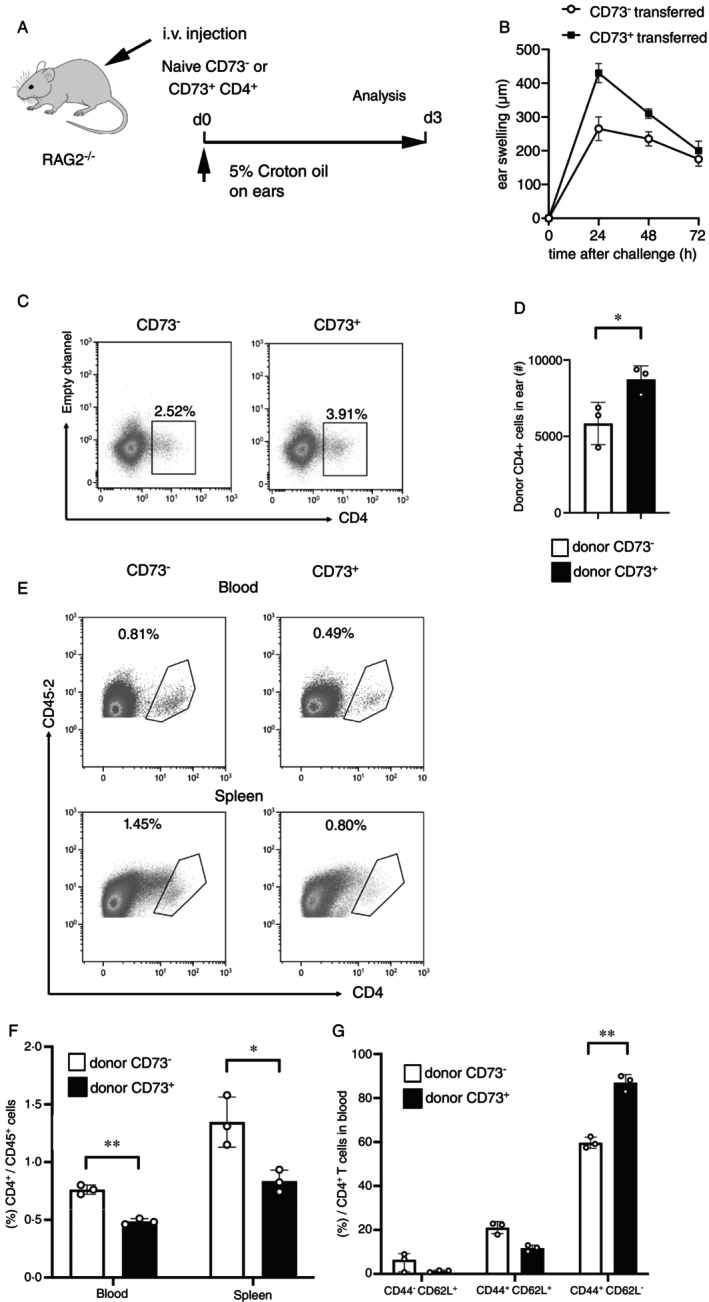
CD73^+^CD4^+^ T cells migrate to inflamed skin. (A) Schematic diagram of the experimental schedule. One hour after transfer of the donor cells, croton oil (1 mg/20 μL) was applied to both ears of the recipient Rag2^−/−^ mice. The ear thickness was monitored over three days. On day 3, mice were euthanized to analyse the transferred cells in blood, spleen and ear by flow cytometry. (B) Ear thickness was measured before and after topical application of croton oil in the recipient Rag2^−/−^ mice. Ear swelling was calculated as ∆ear thickness = ear thickness − baseline thickness. (C + D) On day 3, the number and proportion of the donor CD4^+^ cells within viable CD45^+^ cells in the inflamed ears were determined. Results are shown as representative dot plots (c) and summary data (D). (E + F) The frequency of donor CD4^+^ cells among viable CD45^+^ cells in the blood and spleens was quantified on day 3. Results are shown as representative dot plots (E) and summary data (F). (G) The frequencies of transferred cells expressing naïve (CD44^−^CD62L^+^), T_EM_ (CD44^+^CD62L^−^), and T_CM_ (CD44^+^CD62L^+^) phenotypes were analysed in the blood on day 3. All data represent the mean ± SD (*n* = 3). *p < 0.05; **p < 0.01; ***p < 0.005, students' t‐test (C‐F) or two‐way ANOVA test (B).

To investigate the development of respective CD73^−^ and CD73^+^ T cell subsets in vivo under inflammatory conditions, naïve CD73^+^ and CD73^−^ cells were transferred into Rag2^−/−^ recipient mice, followed by the topical application of croton oil onto the ears to induce skin inflammation (Figure 5A). The changes in ear thickness (Figure 5B) indicate an increased severity of skin inflammation after injection of CD73^+^ T cells, which were accompanied by higher frequencies and numbers of CD73^+^ cells in the inflamed ears as compared to CD73^−^CD4^+^ cells (Figure 5C,D).

Conversely, the presence of donor cells in the bloodstream as well as in the spleens of Rag2^−/−^ mice receiving naïve CD73^+^CD4^+^ cells, was lower as opposed to that in mice receiving naïve CD73^−^CD4^+^ cells (Figure 5E,F). This observed increase in numbers of CD73^+^ cells in the inflamed tissue and their decrease in blood and spleen, corroborate our data obtained in vitro, showing heightened expression of tissue‐recruiting chemokine receptors (e.g., CCR6 and CCR4) and proinflammatory cytokines (e.g., IFN‐γ, TNFα) by FACS‐sorted CD73^+^ T cells. Further flow cytometric analysis of central memory (CD44^+^CD62L^+^) and effector memory (CD44^+^CD62L^−^) phenotypes revealed a predominance of an effector memory phenotype in blood‐derived CD73^+^ donor T cells by day 3 (Figure 5G). These findings indicate a consistent trend in donor CD73^+^ cells towards differentiation into activated cells and subsequent migration to the inflamed skin.

## 3 Discussion

CD73 has widely been characterized for its ectonucleotidase activity. It catalyses the degradation of AMP to adenosine, which has well‐defined immunosuppressive activities [1, 16]. Therefore, the expression of CD73 by immunosuppressive lymphocyte subsets, for example by Tregs, has primarily been studied [17]. Beyond its immunosuppressive role, CD73 has also been implicated in promoting T cell proliferation, as stimulation by anti‐CD73 antibodies has been shown to enhance T cell proliferation [3, 4, 18]. However, according to our unpublished data, this effect is restricted to human CD4^+^ T cells. In mice, anti‐CD73 treatment of freshly isolated CD4^+^ T cells does not affect their proliferation.

In mice, CD4^+^ T cells can be subdivided into CD73^+^ and CD73^−^ subsets at an approximately 1:1 ratio. Already at the stage of recent thymic emigrants (RTE), T cells express CD73 to an even higher level than normal peripheral T helper cells. That declines further from naïve to the memory “state” of the cells. In line with this, it was shown in humans that the level of CD73 positivity declines with age [8].

One explanation for the presence of CD73^+^ and CD73^−^ naïve T cells could be that current markers inadequately distinguish naïve from previously activated cells. However, the presence of both subsets among recent thymic emigrants (Figure S1) argues against this. Alternatively, TCR affinity may influence CD73 expression [19], as higher self‐reactivity could induce CD73 as a protective mechanism either during thymic selection or in the periphery. CD73 expression might also be stochastically regulated, potentially fixed by genetic or epigenetic mechanisms to maintain a functional pool.

Beyond being a marker for RTEs, differential expression of CD73 is functionally relevant in peripheral T cells. That is, CD73^+^ T cells exhibit a higher proliferative capacity and increased production of TNF‐α and IFN‐γ. These findings are consistent with previously published data from patients with inflammatory bowel disease, where CD4^+^CD73^+^ T cells displayed elevated expression of IFN‐γ and *Rorc* compared to their CD73^−^ counterparts [20]. In addition, upregulation of CD45RO in the human CD73^+^ subset indicates an effector memory phenotype, corresponding to the CD44^+^CD62L^−^ memory phenotype observed in CD73^+^ murine T cells described here.

As CD73 is capable of producing adenosine, it is conceivable that its influence on T cell differentiation is mediated by autocrine production of adenosine, acting through adenosine receptors. Cekic et al. [21] have shown that absence of adenosine A2A receptors leads to a decline in the number of naive but not memory T cells, which would indicate that adenosine is important for the maintenance of naïve T cells. Thus, in contrast to our results, CD73^+^, adenosine producing T cells may rather stay naïve as compared to their CD73^−^ counterparts.

Moreover, adenosine has been shown to inhibit the differentiation of naïve T cells into Th1 and Th2 subsets [22, 23], while promoting the development of non‐pathogenic Th17 [24] and regulatory T cells in mice [25]. Furthermore, significantly reduced serum levels of adenosine have been observed in patients with autoimmune diseases [26, 27]. Therefore, CD73‐derived, autocrinely acting adenosine may not explain the functional dichotomy observed between CD73^+^ and CD73^−^ T cells.

In the course of naïve T cell activation, CD73 is upregulated (data not shown), whereby the initially CD73^−^ T cells acquire expression of CD73 after in vitro stimulation with anti‐CD3/anti‐CD28. Therefore, CD73 expression by CD4^+^ T cells serves as a marker for activated CD4^+^ T cells, and the CD4^+^ subset that already expresses CD73 in naïve mice may be pre‐activated. These features qualify naïve CD73^+^CD4^+^ T cells for being a precursor of an early effector phenotype [28]. Early effector cells are thought to give rise to memory T cells at later stages of an inflammation, but a more detailed phenotype and their further development have not been described yet.

In initial studies memory T cells were derived from IFN‐γ‐nonproducing effectors [29, 30], but investigations using adoptive transfer of CD4^+^ T cells from IFN‐γ reporter mice in an LCMV‐specific mouse model indicate that rather IFN‐γ‐producing effector cells give rise to long‐lived memory T cells after LCMV infection [13]. As the memory cell population comprized CD62L^lo^ as well as CD62L^hi^ subsets, these data suggest that both, T_EM_ cells and T_CM_ cells, could be derived from IFN‐γ‐producing precursors [22, 31]. Our results support the notion that IFN‐γ is an important factor for development of memory functions, as the CD73^+^, “memory‐precursor” subpopulation is producing more IFN‐γ than their CD73^−^ counterparts.

Beyond the production of IFN‐γ, our expression analysis of genes relevant for T cell lineage‐commitment, that is, *Ifng* and *Tbx21* and *Cxcr3*, indicate a preference for development into Th1‐like lymphocytes [11], which are prone to give rise to memory cells [13]. Moreover, at the same time reduced levels of *Gata3* and *Rorc*, which encode transcription factors for Th2 and for Th17 differentiation are suppressed. Finally, CD73^+^ T cells sorted by flow cytometry, are more effective to give rise to CD44^+^CD62L^−^ T cells (with CD44 being a key memory marker). Therefore, this supports our notion that CD73^+^ T cells are precursors for development of early effectors, acquiring memory phenotypes later.

IFN‐γ, produced by the CD73^+^ subset, supports the initial starting phase of an immune reaction as it stimulates other immune cells and preferentially supports proinflammatory killer—and Th1 effector T cells. However, in the contraction phase of an inflammation, hyperactivated T cells, which are solely programmed to act as effectors, have to be deleted, without affecting the developing memory T cells. According to our data, CD73^+^CD4^+^T cells seem to be protected from activation induced cell death (AICD), as well as from apoptosis, which are both key mechanisms to get rid of effector cells in the contraction phase of an inflammation [32, 33]. As aberrant contraction phases have been observed in vivo in the absence of IFN‐γ in models for chronic and acute infections, and IFN‐γ deficient cells appear resistant to contraction [34, 35], the elevated production of IFN‐γ by the CD73^+^ subset may therefore sensitize CD73^−^CD4^+^ effector T cells to AICD [36]. In this context, it is of importance that CD73^+^ T cells must be protected from self‐inflicted AICD, possibly induced by autocrinally produced TGF‐β. Therefore, the observed production of TGF‐β (unpublished observation) may be important, as TGF‐β, together with IL‐2, is able to rescue effector cells from apoptosis by heightening the threshold for AICD [37, 38, 39].

When evaluating the role of CD73 in the homeostatic proliferation of naïve CD4^+^ T cells in lymphopenic Rag2^−/−^ mice, we observed a lower frequency of donor CD73^−^ cells compared to CD73^+^ cells, indicating that CD73 expression promotes the survival of CD4^+^ T cells in vivo. Moreover, Ki67 expression was higher in donor CD73^+^ cells, suggesting that CD73 supports the spontaneous proliferation of CD4^+^ T cells under homeostatic conditions. Regarding memory cell differentiation, CD73 promotes the differentiation of naïve CD4^+^ T cells into effector memory cells, which is consistent with in vitro RNA expression data. Observations in human inflammatory bowel disease support our notion, as a Th17 effector memory phenotype is dominant in the CD4^+^CD73^+^ subset of T cells found in blood and the gut [20].

These results are supported by other findings [15], showing that CD73 favours the homeostatic proliferation of CD8^+^ T cells. This is evidenced by a higher frequency of WT CD8^+^ T cells in all organs, including blood, spleen, and lymph nodes, compared to CD73‐deficient CD8^+^ T cells. Additionally, the reduced number of CD73‐deficient CD8^+^ T cells is also accompanied by their reduced Ki67 expression. These findings collectively indicate that CD73 is essential for maintaining the homeostatic proliferation of naïve T cells, including both CD4^+^ and CD8^+^ T cells.

When skin inflammation was induced in Rag2^−/−^ host animals by the application of croton oil, an increased number of CD73^+^ cells were detected in the inflamed ears, accompanied by a higher proportion of effector memory T cells in the spleen, resulting in more severe ear swelling. These findings indicate that CD73 enhances both the activation and migratory capacity of T cells towards inflamed tissues. Similar observations have been reported in a murine experimental autoimmune encephalomyelitis model [40], where CD73^+^ T cells were among the first to infiltrate the brain. In contrast, CD73^−/−^ mice were completely protected from experimental autoimmune encephalomyelitis induction, as T cells failed to enter the cerebrospinal fluid. This latter finding suggests a role for CD73 in mediating adhesion, a hypothesis further supported by a study from Airas et al. [41], showing that binding of lymphocytes to cultured endothelial cells is inhibited by the addition of anti‐CD73 monoclonal antibodies.

While our data indicate that CD73 plays a role in the formation of memory T cells (_TM_) in general [16, 42], it does not appear to specifically support the differentiation of central memory T cells (T_CM_). Instead, effector memory T cells (T_EM_) are more likely to derive from CD73^+^ precursors. This is consistent with human data showing preferential expression of CD73 in T_EM_ cells [20], as well as evidence that CD73^+^ T cells can amplify T cell activation [43]. Moreover, although CD73 is often associated with an immunosuppressive phenotype, subsets of CD73^+^ T cells that are highly reactive and less susceptible to regulatory control have been described in the context of HIV infection [44] and cancer [45]. These are all features that align with the rapid effector responses characteristic of T_EM_ cells. More recently, CD4^+^ and CD8^+^ T cell subsets that do not fall within the traditionally defined categories of central memory (TCM) or effector memory (TEM) cells have been identified as expressing CD73 [8]. Although the frequency of these subsets declines with age in humans, they are long‐lived and exhibit effector functions, characteristics consistent with a memory T cell phenotype. In these cells, CD73 is not merely a surrogate marker of a regulatory network, but rather plays a direct role in promoting T cell survival. These in vivo findings are consistent with our own observations in mice, where CD73^+^ T cells are more resistant to cell death and exhibit higher proliferative capacity compared to their CD73^−^ counterparts.

In many tumour models and inflammatory diseases, the function of CD73 has so far been reduced to its ectonucleotidase activity and to its immune suppressive functions, owed to the production of adenosine. Beyond that, our studies clearly indicate a functional dichotomy of CD73^+^ and CD73^−^ T cell subsets. whereby the CD73^+^ subset contains highly reactive early effector cells, which in the further course of their differentiation give rise to memory T cells. Part of their functions, for example, longevity, proliferation and resistance to AICD, is critically dependent on CD73 expression, as CD4^+^ T cells derived from CD73^−/−^ mice are lacking these features. Because anti‐CD73 antibodies are already in use as checkpoint inhibitors in clinical trials [46], the effects on the CD73^+^ T cells in vivo, beyond the mere blockade of adenosine production, have to be investigated thoroughly. According to our data, blocking CD73 may affect the course of an immune reaction and the development of immune memory.

## 4 Materials and Methods

### 4.1 Mice

C57BL/6 wildtype (WT) mice were purchased from Janvier Labs (Le Genest‐Saint‐Isle, France). CD73‐deficient (CD73^−/−^) mice on the C57BL/6 background and littermates thereof were kindly provided by Prof. Schrader from the University of Düsseldorf, Germany. Rag2‐deficient (Rag2^−/−^) mice were obtained from Jackson (Jackson Labs, Bar Harbour, ME, USA) and tested for T cell absence by flow cytometry. The mice were kept in specific pathogen‐free conditions at the animal facility of Heidelberg University. All animal experiments were carried out under guidelines for animal welfare established by the state of Baden‐Württemberg and were approved by the local ethics review committees. 8–12‐weeks old mice were used for all experiments.

### 4.2 Chemicals, Antibodies, and Flow Cytometry

PBS and croton oil were from Merck (Darmstadt, Germany). FasL (soluble) was purchased from Enzo Life Sciences (New York, the USA). Antibodies including Alexa Fluor 488‐anti‐Foxp3 (MF‐14), APC‐anti‐CD73 (TY/11.8), FITC‐anti‐CD62L (MEL‐14), Brilliant Violet 421‐anti‐CD44 (IM7), Alexa Fluor 488‐anti‐T‐bet (4B10), Brilliant Violet 421‐anti‐GATA3 (16E10A23), PE‐anti‐CD95 (Fas) (SA367H8), APC/Fire 750‐anti‐CD45.2 (104), PE/Cyanine7‐anti‐CD4 (RM4‐4) and Brilliant Violet 421‐anti‐CD25 (PC61) were obtained from Biolegend (Biolegend, Kassel, Germany). APC‐anti‐CD25 (PC61.5), PerCP‐eFluor 710‐anti‐Ki67 (SolA15) and PE‐eFluor 610‐anti‐ROR gamma (t) (B2D) were purchased from Invitrogen (ThermoFischer, Dreieich, Germany). Antibodies for human PBMCs: PerCP‐anti‐CD3 (UCHT1), PE‐anti‐FOXP3 (150D), Brilliant Violet (BV) 421‐anti‐CD45RO (UCHL1), BV510‐anti‐CD27 (O323) were obtained from BioLegend. Brilliant Ultra Violet (BUV) 395‐anti‐CD4 (SK3), BUV496‐anti‐CD8 (RPA‐T8), BUV605‐anti‐IFN‐γ (B27), FITC‐anti‐CD25 (M‐A251), RealBlue 613‐anti‐CCR7 (3D12) antibodies were purchased from BD Bioscience (Heidelberg, Germany). APC‐Cy7‐anti‐CD45RA (HI100) antibody and viability stain LIVE DEAD Fixable Blue were obtained from Invitrogen (ThermoFischer, Dreieich, Germany).

For surface staining, Cells were incubated with respective antibodies in FACS buffer (PBS +3% FCS) for 30 min at 4°C. Intracellular staining if applicable is followed by fixation and permeabilization with eBioscience Intracellular Fixation and Permeabilization buffer (ThermoFisher, Dreieich, Germany). Cells were then stained with antibodies against intracellular proteins in permeabilization buffer for 1 h on ice. Flow cytometry data were collected on a Gallios flow cytometer (Beckman Coulter, Krefeld, Germany) and analysed using Kaluza 2.1 software (Beckman Coulter).

Analysis of human cells: Peripheral blood mononuclear cells (PBMC) were isolated from buffy coats of healthy human donors that had given informed consent. For intracellular cytokine staining, cells were restimulated in a complete RPMI medium containing 10% FBS, 1% Penicillin–Streptomycin, 1% MEM Non‐Essential Amino Acids, 1 mM Sodium Pyruvate, 25 mM HEPES, 50 μM β‐Mercaptoethanol and 2 mM GlutaMAX (Gibco, ThermoFischer, Dreieich, Germany) with phorbol‐12‐myristate‐13‐acetate (PMA) (50 ng/mL), Ionomycin (500 ng/mL), and Brefeldin A (1 μg/mL) at 37°C for 4 h. Fc receptors were blocked with Human TruStain FcX (BioLegend, Koblenz, Germany). Surface and intracellular staining was performed according to standard protocols. Flow cytometry was conducted using the Aurora flow cytometer and SpectroFlo software (Cytek Biosciences, Amsterdam, Netherlands) with further analysis in FlowJo software (TreeStar Inc., Ashland).

### 4.3 Quantitative Reverse Transcription Polymerase Chain Reaction (qRT‐PCR)

Total RNA was extracted from samples using RNeasy plus mini kit based on the manufacturer's protocol (Qiagen, Hilden, Germany). RNA was reversely transcribed to yield cDNA by using RevertAid First Strand cDNA Kit. Real‐time RT‐PCR was performed in a StepOnePlus Real‐Time RT‐qPCR system (all from ThermoFisher, Dreieich, Germany). The 2^−ΔΔCt^ method was used to determine the relative quantities of mRNA expression in comparison with that of Hprt1. Primers were purchased from Eurofins Genomics, (Eurofins, Ebersberg, Germany).

### 4.4 Cytokine Measurement by LEGENDplex Assay and ELISA

Quantification of 13 mouse cytokines including IL‐1α, IL‐1β, IL‐6, IL‐10, IL‐12p70, IL‐17A, IL‐23, IL‐27, MCP‐1, IFN‐γ, IFN‐β, TNF‐α, and GM‐CSF was performed by LEGENDplex Mouse Inflammation Panel (Biolegend, Kassel, Germany), using fluorescence‐encoded beads in flow cytometry.

### 4.5 T Cell Subset Isolation and Sorting

CD3^+^ T cells were enriched by negative selection according to the protocol of Mouse T Cell Isolation Kit (Miltenyi, Bergisch Gladbach, Germany). Cell sorting was performed on the FACSMelody sorter (Becton Dickinson, Heidelberg, Germany), after stained with respective antibodies. CD73^+^CD25^−^ and CD73^−^CD25^−^ T cells were obtained from gating on CD69^−^CD19^−^CD56^−^CD8^−^CD4^+^CD25^−^ T cell populations. Naive CD73^+^ and CD73^−^CD4^+^ T cells, based on CD44^−^CD25^−^CD8^−^CD4^+^ and CD62L^+^ expression followed by gates on CD73^+^ and CD73^−^ cells from negatively isolated CD3^+^ T cells, were sorted for assays using “naïve” CD4^+^ T cells. Sorted cells were collected in sterile polystyrene round‐bottom tube containing 1 mL complete medium. The resulting purified cell populations, with a purity of > 95%, were used for downstream analysis.

### 4.6 Activation and Proliferation of T Cells

96‐well round‐bottom plates (Greiner, Frickenhausen, Germany) coated with anti‐CD3ε antibodies were prepared by adding 100 μL/well of 10 μg/mL solution of anti‐CD3 mAbs in PBS. The coated plates were incubated at 37°C for at least 2 h. The antibody solution was aseptically removed and plates were washed 3 times with sterile PBS.

For the activation and proliferation of T cells, purified CD3^+^ or CD4^+^ T cells were cultured in plate‐bound anti‐CD3 and soluble anti‐CD28 (1 μg/mL) antibodies with 2 × 10^5^ cells in 200 μL complete medium per well, in the presence or absence of IL‐2 (40 U/mL), as indicated. Cells were subsequently cultured at 37°C in a humidified incubator with 5% CO_2_ for 72 h. The proliferation of T cells was measured by ^3^H‐Thymidine (Hartmann Analytics, Braunschweig, Germany) incorporation, using a beta max counter (PerkinElmer, Frankfurt, Germany). In some experiments, the proliferation of T cells was measured with cell proliferation dye eFlour 450 (eBiosciences, Thermofisher, Dreieich, Germany). Briefly, 10 × 10^6^ T cells were labelled with cell proliferation dye eFlour 450 at a concentration of 10 μM. T cells were then incubated with anti‐CD3/anti‐CD28 antibodies as described above, with or without anti‐CD73 mAbs (TY23), in 96‐round‐bottom well plates in 200 μL of complete medium for 3 days at 37°C under 5% CO_2_. At the end of the experimental time course, the dilution of the proliferation dye indicating proliferating cells was quantified by flow cytometry by gating on viable Zombie Aqua^−^ CD4^+^ T cells.

To examine the proliferation of naïve CD4^+^ T cell subtypes induced by bone marrow‐derived dendritic cells (BMDCs), BMDCs were generated from mouse bone marrow with cytokines (10 ng/mL GM‐CSF and 10 ng/mL IL‐4, Miltenyi Biotec) according to standard protocols [33]. BMDCs were co‐cultured with naïve CD4^+^ T cells and plate‐bound anti‐CD3 antibodies in 96‐well plates, and T cell proliferation was assessed after 48 h by ^3^H‐Thymidine incorporation.

### 4.7 CD4 ^+^ T Helper Cells Polarization

The polarization of T cell subtypes was induced by using defined mixtures of cytokines and antibodies. Briefly, for all subtypes of T helper cells polarization, 2 × 10^5^ naïve CD4^+^ T cells were placed in individual well of 96‐well culture dish with plate‐bound anti‐CD3 and soluble anti‐CD28 mAbs as described above. The supplementary cytokines and antibodies are described as below. Briefly, Th1 differentiation is induced with IL‐2 (50 U/mL), IL‐12 (10 ng/mL) and anti‐IL‐4 (10 μg/mL) antibodies; Th2 differentiation with IL‐2 (50 U/mL), IL‐4 (10 ng/mL) and anti‐IFN‐γ (10 μg/mL) antibodies; Th17 differentiation with IL‐6 (50 ng/mL), IL‐1β (10 ng/mL), TGF‐β1 (2 ng/mL), TNF‐α (1 ng/mL), anti‐IFN‐γ (2 μg/mL) and anti‐IL‐4 (2 μg/mL) antibodies; iTreg differentiation with TGF‐β1 (15 ng/mL), IL‐2 (30 U/mL), anti‐IFN‐γ (5 μg/mL) and anti‐IL‐4 (5 μg/mL) antibodies. The cytokines and antibodies are purchased from Biolegend (Kassel, Germany), Miltenyi Biotec (Bergisch Gladbach, Germany) and Peprotech (Hamburg, Germany). After 96 h of incubation, intranuclear staining of lineage‐specific transcription factors was performed to test the efficiency of the polarization with flow cytometry: antibodies against T‐bet and Gata3 were used to examine Th1 and Th2 cells; antibodies against RORγt and Foxp3 were applied to assess Th17 and iTreg cells.

### 4.8 Adoptive Transfer Experiments

Naïve CD73^−^CD4^+^ cells and naïve CD73^+^CD4^+^ were obtained by cell sorting using the BD FACSMelody sorter. The initial input and phenotype were analysed by flow cytometry. A total of 10^6^ cells were intravenously (i.v.) transferred into Rag2^−/−^ mice. On days 6 and 12, mice were euthanized, and the transferred cells were analysed in the blood and spleen by flow cytometry.

To initiate skin inflammation by croton oil, one hour after adoptive transfer of cells into the Rag2^−/−^ mice, croton oil dissolved in acetone (0.8%) was topically applied to the ears (8 μL). Ear thickness was monitored for 3 days to assess the inflammation‐induced ear swelling. On day 3, mice were euthanized to evaluate the distribution and phenotype of the transferred cells in blood, spleen, and ears by flow cytometry.

### 4.9 Statistics

Data were analysed in GraphPad Prism version 10. Comparisons between within two groups was assessed by unpaired Student's *t*‐test or paired *t*‐test. Statistical significance in experiments with more than two groups was analysed by one‐way ANOVA or two‐way ANOVA. GraphPad Prism 10, Kaluza and Microsoft Adobe Illustrator were used to generate diagrams and figures. Different levels of significance are statistically considered as *p < 0.05, **p < 0.01, ***p < 0.001, and ****p < 0.0001, whereas non‐significant *p*‐values are labelled as ‘ns’, or un‐labelled. Differences between groups are shown by a horizontal line with capped end in figures. Data are presented as mean ± SD unless otherwise indicated.

## Author Contributions

L.C. designed and performed experiments, wrote the initial draft of the manuscript; S.R. performed experiments; A.J. performed experiments; F.C.K. designed and analysed experiments; A.E. wrote the paper; K.M. designed experiments and wrote the manuscript.

## Ethics Statement

This study and its experimental procedures were approved by the State of Baden Württemberg. All animal housing and experiments were conducted in accordance with institutional guidelines for the care and use of laboratory animals.

## Conflicts of Interest

The authors declare no conflicts of interest.

## Supporting information


**Figure S1.** Development of CD4^+^CD73^+^ T cells in thymus. (A) Thymic cells were divided according to CD4 and CD8 expression into double positive (DP), double negative (DN), CD4 single positive (CD4 SP) and CD8 single positive (CD8 SP) cells. TCR receptor expression was used to divide cells further into TCRβ low‐, TCRβ intermediate‐ and TCRβ high expressing subsets. CD73 expression is depicted in histograms below, showing appearance of CD73^+^ cells in the TCR high group. (B) CD4^+^SP cells as depicted in (A) are separated into mature and semi‐mature subsets according to their expression of TCRβ and CD24. CD73 expression is depicted in histograms, showing a substantial amount of CD73^+^ T cells in the mature subpopulation. (C) Pregated CD4^+^Lin^−^ (CD19^−^, CD56^−^, MHC class II^−^, CD11b^−^) cells, isolated from peripheral blood, were analysed for recent thymic emigrants (RTEs) according to their CD24 and CD31 expression. Expression of CD73 by CD4^+^ RTEs is shown as dot plot.

**Figure S2.** Expression of CD73 by human T cell populations. (A&B) Definition of T cell populations and gating with exemplary plots showing CD73 expression by human CD4^+^ and CD8^+^ T cells. Dead and CD3‐negative cells were excluded (not shown). Gates for CD73 were set by using FMO controls (not shown). (C) Summary of data from 3 different healthy human donors (PBMCs isolated from buffy coats) showing the percentage of cells expressing CD73 by the indicated population. (D) FACS plots of activated T cell populations (gated as above) with IFN‐γ vs. CD73 expression. (E, F) Statistical analysis of IFN‐γ expression of CD73 positive vs. negative populations, with (E) displaying the percentage of IFN‐γ positive T cells of the respective T cell subpopulation from either CD73^+^ or CD73^−^ populations and (F) displaying the geometric mean intensity (Geo MFI) of the IFN‐γ staining of IFN‐γ^+^ cells of the respective T cell subpopulation from either CD73^+^ or CD73^−^ populations. Data show the mean ± SD (*n* = 3). *p < 0.05; **p < 0.01; students paired t‐test. (G) Exemplary plot of naïve CD4^+^ T cells with IFN‐γ and CD73 expression.

## Data Availability

The data that support the findings of this study are available from the corresponding author upon reasonable request.
