# Supplementary figures and images for: CD73 Expression by CD4 + T Cells Marks Early Effector Memory T Cells
Source: Immunology. 2025 Jun 26;176(4):454–67. doi: 10.1111/imm.70011 (PMC12583237; doi:10.1111/imm.70011)

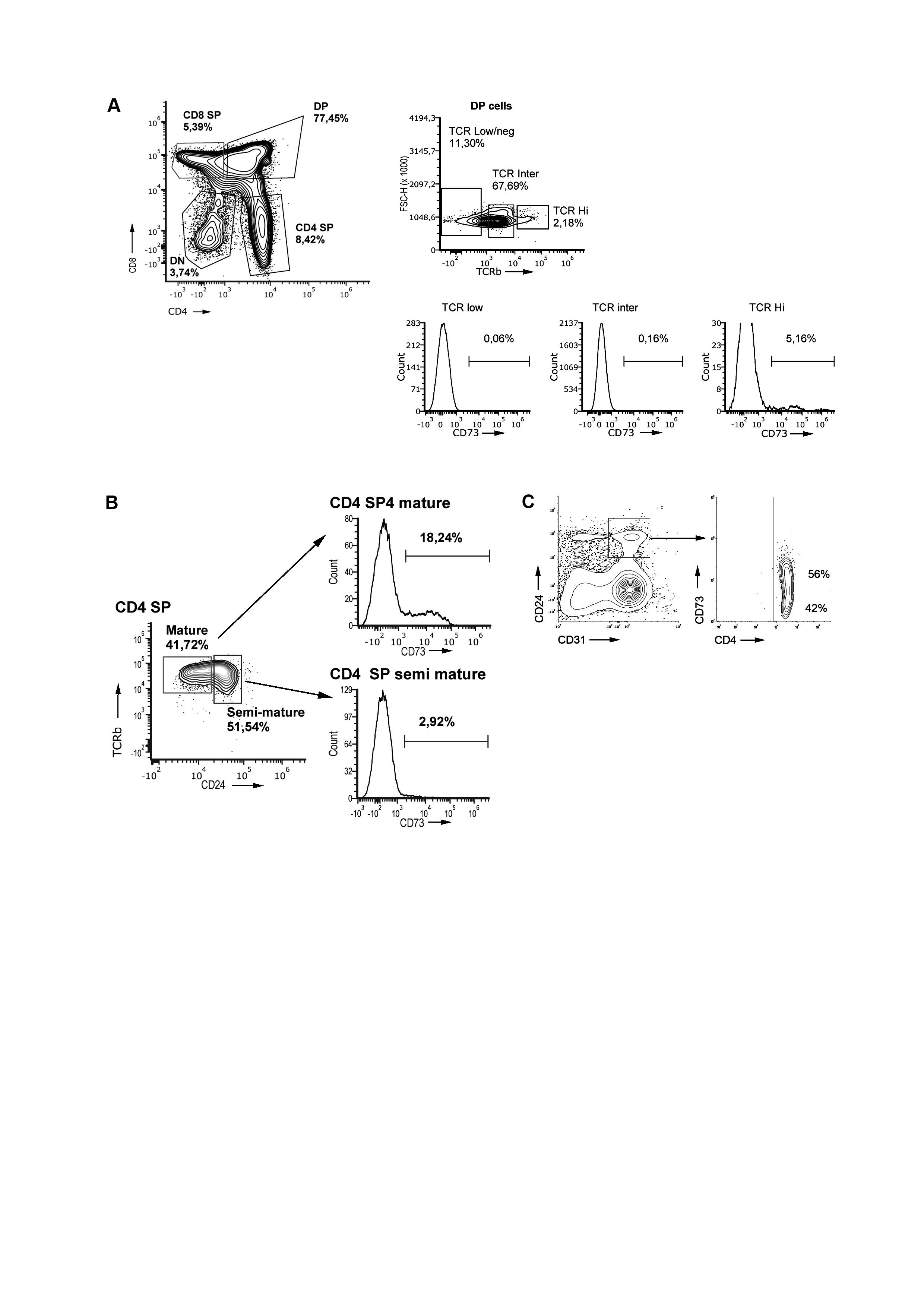

Supplement: Supplementary file 1 — Figure S1. Development of CD4+CD73+ T cells in thymus. (A) Thymic cells were divided according to CD4 and CD8 expression into double positive (DP), double negative (DN), CD4 single positive (CD4 SP) and CD8 single positive (CD8 SP) cells. TCR receptor expression was used to divide cells further into TCRβ low‐, TCRβ intermediate‐ and TCRβ high expressing subsets. CD73 expression is depicted in histograms below, showing appearance of CD73+ cells in the TCR high group. (B) CD4+SP cells as depicted in (A) are separated into mature and semi‐mature subsets according to their expression of TCRβ and CD24. CD73 expression is depicted in histograms, showing a substantial amount of CD73+ T cells in the mature subpopulation. (C) Pregated CD4+Lin− (CD19−, CD56−, MHC class II−, CD11b−) cells, isolated from peripheral blood, were analysed for recent thymic emigrants (RTEs) according to their CD24 and CD31 expression. Expression of CD73 by CD4+ RTEs is shown as dot plot. [file IMM-176-454-s002.jpg]

Supplemental Figure S2

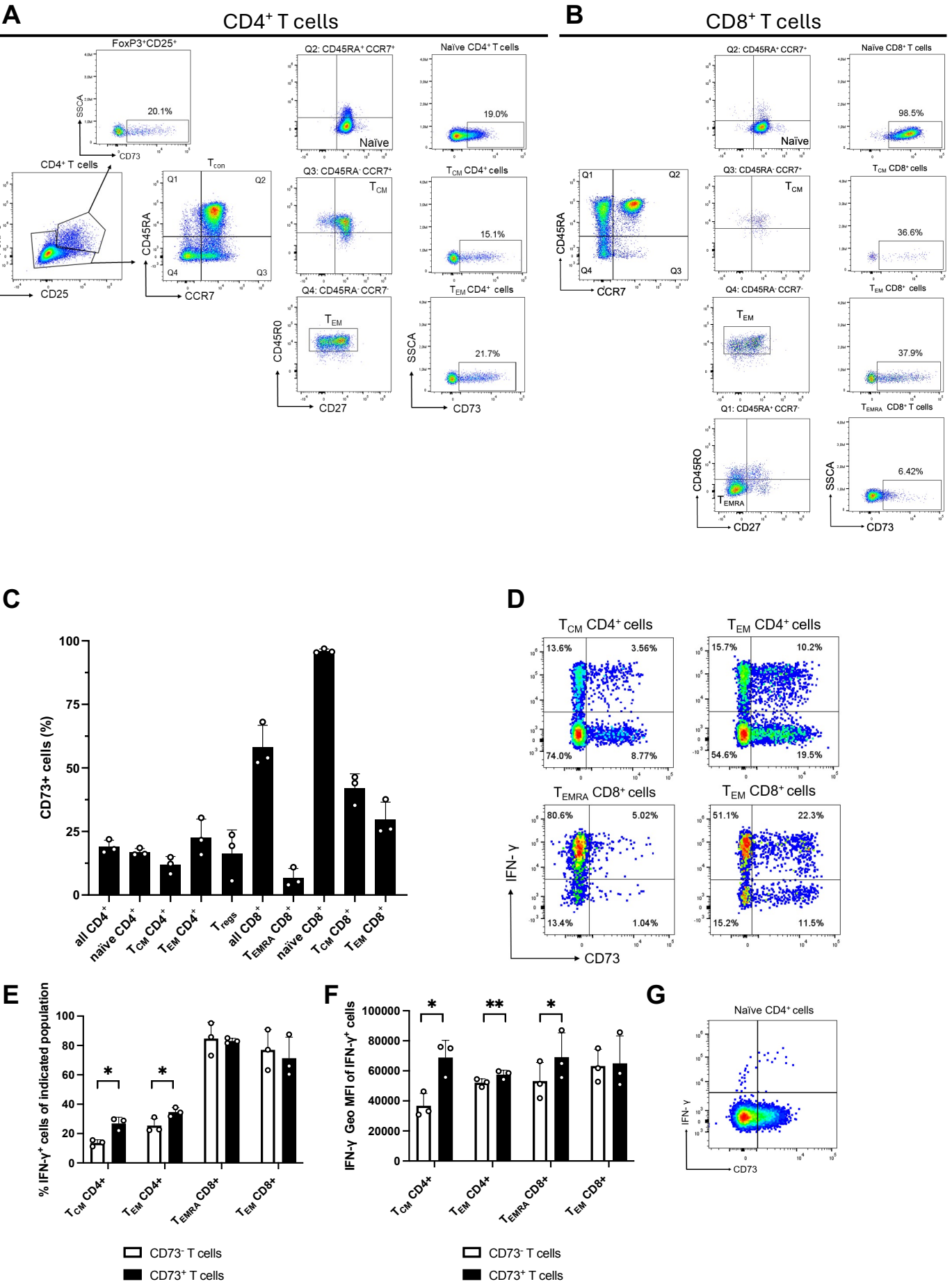

Supplement: Supplementary file 2 — Figure S2. Expression of CD73 by human T cell populations. (A&B) Definition of T cell populations and gating with exemplary plots showing CD73 expression by human CD4+ and CD8+ T cells. Dead and CD3‐negative cells were excluded (not shown). Gates for CD73 were set by using FMO controls (not shown). (C) Summary of data from 3 different healthy human donors (PBMCs isolated from buffy coats) showing the percentage of cells expressing CD73 by the indicated population. (D) FACS plots of activated T cell populations (gated as above) with IFN‐γ vs. CD73 expression. (E, F) Statistical analysis of IFN‐γ expression of CD73 positive vs. negative populations, with (E) displaying the percentage of IFN‐γ positive T cells of the respective T cell subpopulation from either CD73+ or CD73− populations and (F) displaying the geometric mean intensity (Geo MFI) of the IFN‐γ staining of IFN‐γ+ cells of the respective T cell subpopulation from either CD73+ or CD73− populations. Data show the mean ± SD (n = 3). *p < 0.05; **p < 0.01; students paired t‐test. (G) Exemplary plot of naïve CD4+ T cells with IFN‐γ and CD73 expression. [file IMM-176-454-s001.pdf]
